# Complete Genome Sequence of *Dyella jiangningensis* Strain SBZ3-12, Isolated from the Surfaces of Weathered Rock

**DOI:** 10.1128/genomeA.00416-14

**Published:** 2014-05-15

**Authors:** Yuanyuan Bao, Amy Ho Yan Kwok, Linyan He, Jingwei Jiang, Zhi Huang, Frederick Chi-ching Leung, Xiafang Sheng

**Affiliations:** a Key Laboratory of Agricultural and Environmental Microbiology, Ministry of Agriculture, College of Life Sciences, Nanjing Agricultural University, Nanjing, China; b Bioinformatics Center, Nanjing Agricultural University, Nanjing, China

## Abstract

*Dyella jiangningensis* strain SBZ3-12 can weather biotite and release Al and Fe from biotite under nutrient-poor conditions. Here, we report the first complete genome sequence of *D. jiangningensis* strain SBZ3-12, which may facilitate a better understanding of the molecular mechanism behind mineral weathering.

## GENOME ANNOUNCEMENT

Strain SBZ3-12 was isolated from surfaces of weathered mineral rocks using the dilution plating method. Based on our previous phenotypic, chemotaxonomic, and phylogenetic analysis, it is proposed that strain SBZ3-12 represents a novel species of the genus *Dyella*, which is designated *Dyella jiangningensis* sp. nov. (1). Mineral weathering experiments showed that strain SBZ3-12 was able to weather biotite and released more Al and Fe than the control. Despite decades of dedicated efforts by many scholars, controversies remain regarding the roles of microorganisms in mineral precipitation and weathering and the molecular mechanism behind the bio-degradation (2, 3). Here we present the whole-genome sequence of *D. jiangningensis* strain SBZ3-12.

Genomic DNA of *D. jiangningensis* strain SBZ3-12 was isolated following the methods listed in Wilson’s study (4). The quality of DNA was examined using a NanoDrop2000 spectrophotometer (Thermo Scientific). The SBZ3-12 genome was sequenced using a 454 GS Junior pyrosequencing system (Roche) with ~25-fold coverage. One shotgun-genome run and one 8-kb library span paired-end run were conducted, yielding 373,571 reads. These reads were assembled into 39 large contigs (length, >500 bp) using the Newbler 2.4 assembly software program (Roche). Gaps between large contigs were filled by Sanger sequencing of the PCR products by means of an ABI 3730XL capillary sequencer and subsequent assembly using SeqMan software (DNASTAR). The open reading frames (ORFs) and rRNA and tRNA genes were annotated by the NCBI Prokaryotic Genome Automatic Annotation Pipeline (PGAAP) (5). Functional classification was performed by aligning predicted proteins to the Clusters of Orthologous Groups (COG) database (6). All predicted genes were compared to a nonredundant (nr) protein database in NCBI using BLASTX (7), with *E* values of 1 × 10^−5^ and filtering at 20% match identity and 90% alignment length. Metabolic pathways were analyzed by a bi-directional best-hit method on the KEGG web server (8).

*D. jiangningensis* strain SBZ3-12 has one chromosome, which is 5,396,991 bp in size with a G+C content of 64.22%. The chromosome has 4,666 predicted coding sequences (CDS), and 59 RNAs were identified, including 52 tRNAs, 6 rRNAs, and 1 non-coding RNA (ncRNA). As for the chromosome genes, 61.23% were assigned to specific COGs, and approximately 74.07% of the genes were assigned to a KEGG orthologous number and were involved in 202 predicted metabolic pathways. Moreover, 36 CDS encoding outer membrane receptor proteins (involved mostly in Fe transport) were annotated and may play important roles in mineral weathering (9).

### Nucleotide sequence accession number.

The complete genome sequence of *D. jiangningensis* strain SBZ3-12 has been deposited at DDBJ/EMBL/GenBank database under the accession no. CP007444. The version described in this paper is the first version.

## References

[1] Zhao F Guo XQ Wang P He LY Huang Z Sheng XF 2013 *Dyella jiangningensis* sp. nov., a γ-proteobacterium isolated from the surface of potassium-bearing rock. Int. J. Syst. Evol. Microbiol. 63:3154–3157. 10.1099/ijs.0.048470-0 23435246

[2] Banks ED Taylor NM Gulley J Lubbers BR Giarrizzo JG Bullen HA Hoehler TM Barton HA 2010 Bacterial calcium carbonate precipitation in cave environments: a function of calcium homeostasis. Geomicrobiol. J. 27:444–454. 10.1080/01490450903485136

[3] Petsch ST Eglington TI Edwards KJ 2001 14C-dead living biomass: evidence for microbial assimilation of ancient organic carbon during shale weathering. Science 292:1127–1131. 10.1126/science.1058332 11283356

[4] Wilson K 2001 Preparation of genomic DNA from bacteria. Curr. Protoc. Mol. Biol. 2001:2.4.1–2.4.5 10.1002/0471142727.mb0204s56 18265184

[5] Angiuoli SV Gussman A Klimke W Cochrane G Field D Garrity G Kodira CD Kyrpides N Madupu R Markowitz V Tatusova T Thomson N White O 2008 Toward an online repository of standard operating procedures (SOPs) for (meta)genomic annotation. Omics 12:137–141 10.1089/omi.2008.0017 18416670 PMC3196215

[6] Tatusov RL Galperin MY Natale DA Koonin EV 2000 The COG database: a tool for genome-scale analysis of protein functions and evolution. Nucleic Acids Res. 28:33–36 10.1093/nar/28.1.33 10592175 PMC102395

[7] Altschul SF Madden TL Schäffer AA Zhang J Zhang Z Miller W Lipman DJ 1997 Gapped BLAST and PSI-BLAST: a new generation of protein database search programs. Nucleic Acids Res. 25:3389–3402 10.1093/nar/25.17.3389 9254694 PMC146917

[8] Moriya Y Itoh M Okuda S Yoshizawa AC Kanehisa M 2007 KAAS: an automatic genome annotation and pathway reconstruction server. Nucleic Acids Res. 35:W182–W185 10.1093/nar/gkm321 17526522 PMC1933193

[9] Ferret C Sterckeman T Cornu JY Gangloff S Schalk IJ Geoffroy VA 24 2 2014 Siderophore-promoted dissolution of smectite by fluorescent *Pseudomonas*. Environ. Microbiol. Rep. 10.1111/1758-2229.12146 25646536

